# A familial case of Galloway-Mowat syndrome due to a novel *TP53RK* mutation: a case report

**DOI:** 10.1186/s12881-018-0649-y

**Published:** 2018-07-27

**Authors:** Hye Sun Hyun, Seong Heon Kim, Eujin Park, Myung Hyun Cho, Hee Gyung Kang, Hyun Soon Lee, Noriko Miyake, Naomichi Matsumoto, Hiroyasu Tsukaguchi, Hae Il Cheong

**Affiliations:** 1 Department of Pediatrics, Seoul National University Children’s Hospital, 101 Daehak-Ro, Jongno-Gu, Seoul, 03080 South Korea; 2 Department of Pediatrics, Pusan National University Children’s Hospital, Yangsan, South Korea; 3 Research Coordination Center for Rare Diseases, Seoul National University Hospital, Seoul, South Korea; 4 Renal Pathology Lab, Hankook Kidney and Diabetes Institute, Seoul, South Korea; 5 Department of Human Genetics, Yokohama City University Graduate School of Medicine, Yokohama, Japan; 6 Second Department of Internal Medicine, Kansai Medical University, Osaka, Japan; 7 Department of Pediatrics, Seoul National University College of Medicine, Seoul, South Korea; 8 Kidney Research Institute, Medical Research Center, Seoul National University College of Medicine, Seoul, South Korea

**Keywords:** Galloway–Mowat syndrome, KEOPS complex, *TP53RK* mutation

## Abstract

**Background:**

Galloway–Mowat syndrome (GAMOS) is a rare hereditary renal–neurological disease characterized by early-onset steroid-resistant nephrotic syndrome in combination with microcephaly and brain anomalies. Recently, novel causative mutations for this disease have been identified in the genes encoding the four KEOPS subunits: *OSGEP*, *TP53RK*, *TPRKB,* and *LAGE3*.

**Case presentation:**

We detected a novel homozygous *TP53RK* mutation (NM_033550, c.194A > T, p.Lys65Met) using whole exome sequencing in a familial case of GAMOS with three affected siblings. All three patients manifested similar phenotypes, including very early-onset nephrotic syndrome (8 days, 1 day, and 1 day after birth, respectively), microcephaly, dysmorphic faces, and early fatality (10 months, 21 days, and 25 days of age, respectively). One patient also showed hiatal hernia with gastric volvulus. Renal biopsy performed on one patient revealed focal segmental glomerulosclerosis with severe tubulo-interstitial changes.

**Conclusion:**

We report on a familial case of GAMOS with three affected siblings carrying a novel homozygous *TP53RK* mutation. To our knowledge, this is only the second report on GAMOS in association with a *TP53RK* mutation.

## Background

Galloway-Mowat syndrome (GAMOS) is a rare hereditary renal–neurological disease characterized by early-onset steroid-resistant nephrotic syndrome combined with microcephaly and brain anomalies [1, 2]. There have been several reports of candidate genes for this disease, namely *WDR73* [3–8], *NUP107* [9], and *WHAMM* [10]. Recently, Hildebrandt and colleagues [11] identified novel causative mutations in the genes encoding the four KEOPS (Kinase, Endopeptidase and Other Proteins of small Size) subunits, *OSGEP*, *TP53RK*, *TPRKB,* and *LAGE3*, in 37 individuals from 32 families with GAMOS. They also revealed that knocking down genes encoding KEOPS subunits in human podocytes results in impaired cell proliferation, translational attenuation, endoplasmic reticulum (ER) stress, activation of DNA damage response (DDR) signaling, increased apoptosis, and defects in actin regulation, which are possible pathogenic features of GAMOS [11]. Another study independently reported a familial case of GAMOS with a homozygous *OSGEP* mutation [12].

Here, we report the identification of a homozygous *TP53RK* mutation in a familial case of GAMOS with three affected siblings.

## Case presentations

The parents of the patients were nonconsanguineous and had four offspring (II-1–II-4), three of which exhibited very similar phenotypes (Table 1). This study was approved by the Seoul National University Hospital’s Institutional Review Board (IRB No. 0812–002-264). Informed consent was obtained from the parents.

**Table 1:** Clinical features of affected individuals

Case	II-1	II-2	II-4
Gender	Female	Male	Male
Age of death	10 months	21 days	25 days
Ethnicity	Korean	Korean	Korean
Neonatal Profile			
Gestational period (weeks)	39^+ 3^	36^+ 6^	36^+ 4^
Apgar score (5 min)	7	7	7
Pregnancy course	fetal distress	fetal distress, C/S	fetal distress, C/S
Birth height (cm)	46 (5–10th percentile)	47.5 (10–25th percentile)	45 (3rd–5th percentile)
Birth weight (g)	2250 (< 3rd percentile)	1960 (< 3rd percentile)	1780 (< 3rd percentile)
Head circumference (cm)	29 (< 3rd percentile)	28 (< 3rd percentile)	27 (< 3rd percentile)
Renal Phenotypes			
Onset of nephrotic syndrome	8 days	1 day	1 day
Renal biopsy (age)	FSGS (2 weeks)	NP	NP
Urinary tract abnormalities	(−)	(−)	(−)
Neurological Features			
Brain MRI	microcephaly simplified gyral pattern progressive brain atrophy	microcephaly simplified gyral pattern cerebellar hypoplasia pontine hypoplasia	microcephaly simplified gyral pattern
Cerebellum atrophy	(−)	(−)	(−)
Others	facial dysmorphism hiatal hernia gastric volvulus	facial dysmorphism skeletal deformities	facial dysmorphism

*C/S* Caesarean section, *MRI* magnetic resonance imaging, *NP* not performed

### Case II-1

**Fig. 1:**
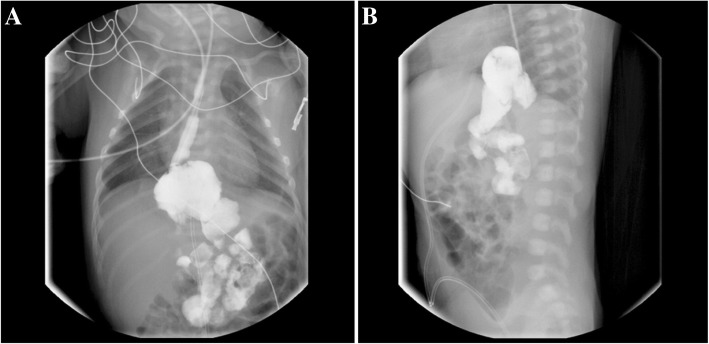
Upper gastrointestinal examination of Case II-1. Anteroposterior (**a**) and lateral (**b**) projections revealed an upward dislocation of the stomach into the mediastinum, which is compatible with a hiatal hernia

**Fig. 2:**
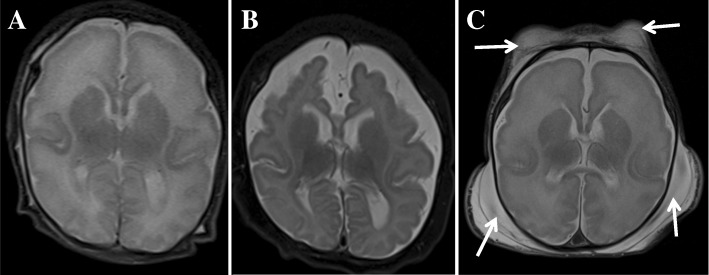
Patients’ brain magnetic resonance images (MRIs). Axial T2-weighted MRI (**a**) of Case II-1 taken at 2 weeks of age shows a simplified gyral pattern with too few and shallow sulci and normal cortical thickness. Follow-up axial T2-weighted MR image (**b**) acquired at 4 months of age shows a progression of diffuse brain atrophy with subarachnoid space widening. Axial T2-weighted MRI (**c**) of Case II-2 obtained at 3 weeks of age shows a similar pattern of simplified sulcation as the sibling. Note extensive fluid collection in the scalp (arrows)

**Fig. 3:**
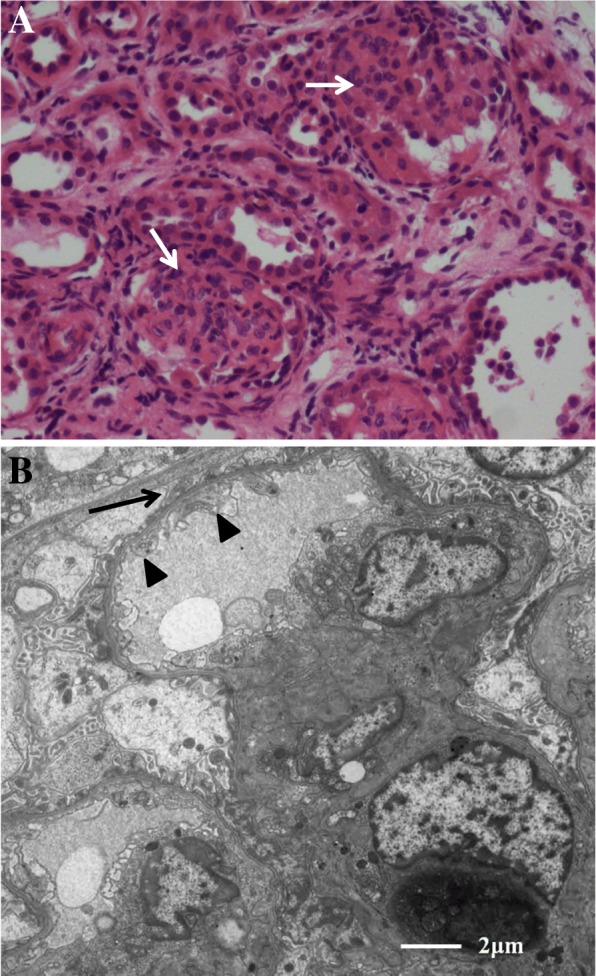
Renal pathological findings from Case II-1 at 2 weeks of age. **a** About half of the glomeruli showed segmental sclerosis (arrows). Hematoxylin and eosin staining, original magnification × 400. **b** Ultrastructurally, the glomerular basement membranes exhibit normal thickness, but show focal subendothelial widening (arrowheads). No electron-dense deposits were found. Epithelial cell foot processes show focal marked effacement (arrows). Original magnification × 5000

This female baby was born after a gestational period of 39^+ 3^ weeks. The birth weight was 2250 g (< 3rd percentile), height was 46 cm (5–10th percentile), head circumference was 29 cm (< 3rd percentile), and the Apgar scores at 1 and 5 min were 5 and 7, respectively. A diaphragmatic hernia was noted in the delivery room. The initial serum albumin level at day one was 3.3 g/dL and the initial serum creatinine level, which reflects the mother’s renal function, was 0.58 mg/dL. Imaging revealed a hiatal hernia with gastric volvulus (Fig. 1). She also had facial dysmorphism including ocular hypertelorism and low-set ears, and a brain magnetic resonance image (MRI) revealed microcephaly with a simplified gyral pattern (Fig. 2a). Surgical repair of the hiatal hernia was performed 6 days after birth without any serious events. The baby started to feed on a mix of breast milk and formula. Two days after surgery, generalized edema developed with a decrease in urine volume. Serum albumin levels decreased to 2.0 g/dL, serum creatinine levels increased to 1.29 mg/dL, and 24-h urinary protein excretion was 2871 mg/day. Renal ultrasonography revealed increased echogenicity of both kidneys with poor differentiation between the peripheral cortex and central echogenic complex. At the age of 2 weeks, an open kidney biopsy was performed (Fig. 3). Thirty-eight (44%) of 87 glomeruli exhibited segmental lobular collapse and sclerosis, and some of the nonsclerotic glomeruli showed features of immature fetal glomeruli. Tubules displayed severe focal atrophy and loss. Infiltration of mononuclear cells and fibrosis were observed in the interstitium. A follow-up brain MRI at 4 months of age showed the progression of diffuse brain atrophy with subarachnoid space widening (Fig. 2b). Massive proteinuria persisted and serum creatinine levels began to rise rapidly at the age of 9 months. However, the baby received conservative treatment only, including intermittent albumin replacement, because the parents did not want immunosuppressive treatment or any other aggressive renal replacement therapy. The baby died at the age of 10 months.

### Case II-2

This male baby was born by cesarean section due to fetal distress at the gestational age of 36^+ 6^ weeks. Birth weight was 1960 g (< 3rd percentile), height was 47.5 cm (10–25th percentile), head circumference was 28 cm (< 3rd percentile), and the Apgar scores at 1 and 5 min were 6 and 7, respectively. The baby displayed various features of facial dysmorphism (microcephaly, almond-shaped eye, abnormally high but narrow forehead, ocular hypertelorism, depressed nasal bridge, large and low-set ears, micrognathia, and high-arched cleft palate), and skeletal deformities (arachnodactyly and clasped thumb). Massive proteinuria, hypoalbuminemia, and generalized edema were noted immediately after birth. Renal ultrasonography revealed bilaterally small echogenic kidneys with poor cortico-medullary differentiation. A brain MRI revealed microlissencephaly, small cerebellar hemisphere size, and decreased volume of the ventral pons (Fig. 2c). At the age of 15 days, serum albumin and creatinine levels were 2.6 g/dL and 2.88 mg/dL, respectively. The parents did not want any active treatment at this time, and the patient died at the age of 21 days due to rapidly decreasing renal function.

### Case II-3

Cases II-3 and II-4 were dizygotic twin babies born after in vitro fertilization. They were born by cesarean section after a gestational period of 36^+ 4^ weeks. The birth weight of Case II-3 was 2830 g (10–25th percentile), height was 46 cm (5–10th percentile), and head circumference was 34 cm (25–50th percentile). Physical examination revealed no abnormalities, and there were no perinatal or neonatal problems. At the age of 6 weeks, serum albumin and creatinine levels were 4.1 g/dL and 0.22 mg/dL, respectively. Urinalysis revealed no proteinuria.

### Case II-4

This male baby was the second of the dizygotic twins. Intrauterine growth retardation was detected by fetal ultrasonography. Birth weight was 1780 g (< 3rd percentile), height was 45 cm (3rd–5th percentile), head circumference was 27 cm (< 3rd percentile), and the Apgar scores at 1 and 5 min were 4 and 7, respectively. He exhibited ocular hypertelorism, micrognathia, and low-set ears. A brain MRI revealed lissencephaly. Generalized edema with massive proteinuria developed soon after birth. Serum creatinine levels at 3 days and 7 days after birth were 1.11 mg/dL and 1.27 mg/dL, respectively. Renal ultrasonography performed at 1 week after birth revealed small echogenic kidneys with poor differentiation between the peripheral cortex and the central echogenic complex. The patient was treated conservatively and died 25 days after birth.

### Mutational analyses

Whole exome sequencing was performed for Case II-2 as previously described [13], which revealed a novel homozygous c.194A > T (p.Lys65Met) mutation in exon 1 of the *TP53RK* gene (NM_033550). This was later confirmed using traditional Sanger sequencing. A study of all family members using Sanger sequencing revealed that Case II-4 carried the same homozygous mutation, and that both parents and Case II-3 were heterozygous for the mutation. Although we were not able to test Case II-1 due to a lack of available samples, her phenotypes were very similar to those of the other two affected siblings who carried the mutation.

## Discussion and conclusions

GAMOS is a heterogeneous disease, both phenotypically and genetically. Phenotypically, in addition to major findings of early-onset nephrotic syndrome and microcephaly, most patients have facial dysmorphia including hypertelorism, ear abnormalities, and micrognathia. Some patients may present arachnodactyly, visual impairment, or other manifestations [9].

Colin et al. [3] identified 2 different truncating mutations in the *WDR73* gene in 3 patients from 2 unrelated families with GAMOS. The authors suggested that WDR73 plays a role in regulating the microtubule network during the cell cycle, and loss of its function leads to impaired neuronal growth and brain development, as well as impaired podocyte growth and maintenance in the kidneys. After the investigation by Colin et al. [3], several studies have reported on additional cases of GAMOS in association with *WDR73* mutations [4–8]. In one of those studies [6], *WDR73* mutations were found in 3 (5.9%) of the 51 unrelated patients with cerebellar atrophy and variable brain anomalies, and in 2 (5%) of the 40 unrelated patients with a clinical diagnosis of GAMOS. Rosti et al. [9] detected *NUP107* mutations in 5 Turkish patients with GAMOS-like phenotypes from two consanguineous families. However, in other reports [14, 15], *NUP107* mutations were associated with isolated nephrotic syndrome. Therefore, there may be an allele-specific critical role for *NUP107* in regulating brain growth and GAMOS-like presentation [9].

Recently, a large-scale study [11] identified novel causative mutations in one of the four genes encoding KEOPS subunits, *OSGEP*, *TP53RK*, *TPRKB* and *LAGE3*, in 37 individuals (from 32 families) out of 91 individuals with GAMOS. In that study, *OSGEP* mutations were the most common, and were detected in 28 individuals from 24 families, while mutations in *TP53RK* were detected in only 4 individuals from 3 families. Almost half of the families carrying *OSGEP* mutations were of Taiwanese or East Asian descent, while the Korean family described in the present case study carried a *TP53RK* mutation. In addition, an independent study [12] reported on a familial case of GAMOS with *OSGEP* mutations. Currently, the five genetic subtypes of GAMOS are listed in the Online Mendelian Inheritance in Men (https://www.ncbi.nlm.nih.gov/omim/) database. GAMOS1–5 are associated with mutations in *WDR73*, *LAGE3*, *OSGEP*, *TP53RK*, and *TPRKB*, respectively. All subtypes exhibit autosomal recessive inheritance, except for GAMOS2, which exhibits X-linked recessive inheritance.

The *TP53RK* mutation identified in the present study [i.e., c.194A > T (p.Lys65Met)] is novel. Co-segregation of the recessive mutation with the phenotype in the family members, including both parents, was confirmed. Interestingly, a known variation involving the same Lys65 [i.e., c.194A > G (p.Lys65Arg)] has a reported allele frequency of 0.00009747 (ExAC Browser, http://exac.broadinstitute.org/). Although we did not perform functional studies, both variations are predicted to be pathogenic according to the web-based program MutationTaster (http://www.mutationtaster.org/). Whole exome sequencing performed for Case II-2 revealed no pathogenic variants in other known candidate genes of GAMOS, namely *WDR73*, *NUP107*, *WHAMM*, and other genes encoding KEOPS subunits. In our whole exome sequencing pipeline, common nucleotide variants (minor allele frequency > 1%) were removed in the middle of the analysis. The only variant in other known proteinuria/focal segmental glomerulosclerosis-associated genes, which was detected by the whole exome sequencing, was a heterozygous c.449–26C > CT (rs35640989) in the *NUP107* gene. Although a mutational study for Case II-1 was not performed owing to a lack of samples, all the three affected babies included in this study showed common manifestations of very early-onset nephrotic syndrome, microcephaly, dysmorphic faces, and early fatal outcome. Case II-1 had an additional finding of a hiatal hernia with gastric volvulus. A hiatal hernia was one of the features observed in the original cases as reported by Galloway and Mowat [1]. However, there have been only few case reports of GAMOS associated with hiatal hernias, including one patient with *WDR73* mutations [6] and a familial case with *OSGEP* mutations [11]. Case II-2 in the present study displayed additional skeletal deformities, including arachnodactyly and clasp thumb. Skeletal deformities are more commonly reported than hiatal hernias in patients with GAMOS [11, 16–18]. In a large study conducted by Braun et al. [11], most of the Taiwanese patients had arachnodactyly and carried *OSGEP* mutations. All three patients in the present study had an early fatal outcome; therefore, we could not evaluate long-term clinical changes in growth and development. To date, only four patients with GAMOS from three families have been reported in association with *TP53RK* mutations [11]. Among those four patients, two sibling cases showed similar phenotypes to those of our patients, i.e., early-onset proteinuria at 2 months of age in both cases, early fatal outcome at 2.5 months and 11 months of age, respectively, and facial dysmorphism with skeletal abnormalities in one case. The other two patients showed relatively later onset at 1 year and 10 months of age, respectively, and later fatal outcomes at 2.5 years and 3 years of age, respectively.

The KEOPS complex contains five subunits: LAGE3, OSGEP, TP53RK, TPRKB, and the recently identified C14orf142 [19]. Braun et al. [10] identified and meticulously studied multiple mutations in the genes encoding the four KEOPS subunits, including *OSGEP*, *TP53RK*, *TPRKB,* and *LAGE3*. The authors conducted short hairpin RNA (shRNA)-mediated knockdown of KEOPS subunits and showed that mutations in *OSGEP*, *TP53RK*, and *TPRKB* could not rescue proliferation defects in human podocytes, suggesting that these alleles impaired protein functionality. Co-immunoprecipitation experiments revealed that the p.Lys60Serfs*61 and p.Thr81Arg mutations in *TP53RK* detected in a family abrogated the interaction between TPRKB and TP53RK [11]. However, other mutations in any of the four genes did not abrogate intermolecular interactions. The canonical function of the KEOPS complex is to mediate *N*^6^-threonylcarbamoyladenosine modifications, known as a t^6^A modification, at position 37 of all tRNAs that recognize codons that start with adenosine, i.e., ‘ANN codons’ [20–23]. Braun et al. [11] demonstrated that knocking down *OSGEP*, *TP53RK,* or *TPRKB* using shRNA in human podocytes resulted in various effects, including decreased t^6^A levels, inhibition of nascent protein synthesis, decreased cell proliferation, activation of the unfolded protein response with ER stress, upregulation of the ER-associated proteasomal degradation system, and increased apoptosis associated with activation of the DDR. Knockdown of one of these three components of the KEOPS complex also disrupts the formation of the sublamellar actin network in human podocytes and decreases podocyte migration [11]. Therefore, the authors concluded that mutations in the KEOPS complex impair both the canonical and noncanonical functions of the KEOPS complex, which would play a major pathogenic role in the development of GAMOS [11].

In conclusion, we report a familial case of GAMOS associated with a novel homozygous *TP53RK* mutation (p.Lys65Met). Three of the four children in the family were affected and displayed similar phenotypes, including very early-onset nephrotic syndrome, microcephaly, facial dysmorphia, and early fatality. In addition, renal biopsy revealed focal segmental glomerulosclerosis in one patient.

## Abbreviations

DDR: DNA damage response
ER: Endoplasmic reticulum
GAMOS: Galloway–Mowat syndrome
KEOPS: Kinase, endopeptidase and other proteins of small size
MRI: Magnetic resonance imaging
